# Critical review of clinical data and expert-based recommendations for the use of bosutinib in the treatment of chronic myeloid leukemia

**DOI:** 10.3389/fonc.2024.1405467

**Published:** 2024-08-26

**Authors:** Valentín García-Gutiérrez, María Teresa Gómez-Casares, Blanca Xicoy, Felipe Casado-Montero, Guillermo Orti, Pilar Giraldo, Juan Carlos Hernández-Boluda

**Affiliations:** ^1^ Hospital Universitario Ramón y Cajal, Irycis, Universidad de Alcalá, Madrid, Spain; ^2^ Servicio de Hematología, Hospital Universitario de Gran Canaria Dr. Negrin. Profesor asociado de la ULPGC, Las Palmas de Gran Canaria, Spain; ^3^ Institut Català d’Oncologia, Hospital Germans Trias i Pujol, Josep Carreras Leukemia Research Institute, Universitat Autònoma de Barcelona, Barcelona, Spain; ^4^ Servicio de Hematología y Hemoterapia, Hospital General Universitario de Toledo, Toledo, Spain; ^5^ Servicio de Hematologia, Hospital Universitari Vall d’Hebron, Vall d’Hebron Institute of Oncology (VHIO), Barcelona, Spain; ^6^ Hematologia, Hospital Quironsalud, Fundación ZeroLMC, Zaragoza, Spain; ^7^ Hospital Clínico Universitario de Valencia (INCLIVA), Universidad de Valencia, Valencia, Spain

**Keywords:** chronic myeloid leukemia, tyrosine kinase inhibitors, bosutinib, adverse event, real world evidence

## Abstract

Chronic myeloid leukemia (CML), characterized by the presence of the *BCR::ABL1* fusion gene, has undergone a transformative shift with the introduction of tyrosine kinase inhibitors (TKIs). The current availability of six different TKIs (imatinib, dasatinib, nilotinib, bosutinib, ponatinib, and asciminib) in clinical practice makes it important to know their efficacy and toxicity profile for treatment optimization. This review examines the latest insights regarding the use of bosutinib in CML treatment. Clinical trials have demonstrated the effectiveness of bosutinib, positioning it as a first-line treatment that can induce sustained molecular responses. Importantly, it can also be effective in patients who have experienced treatment failure or intolerance with prior TKIs, revealing the potential of bosutinib also in second- and later-line settings. Even in the advanced phase of CML, bosutinib has demonstrated its capacity to achieve molecular responses, expanding its usefulness. Real-world evidence studies echo these findings, emphasizing bosutinib’s effectiveness in achieving deep molecular responses, maintaining remissions, and serving as an alternative for patients intolerant or resistant to other TKIs as a second-line therapy. Notably, one of the greatest strengths of bosutinib is its favorable safety profile, in particular the low incidence of vascular complications with its use, which is undoubtedly a comparative advantage over other TKIs. In summary, the latest research highlights the versatility of bosutinib in CML treatment and underscores its pivotal role in optimizing patient management in challenging cases. Continuing research and investigation will further establish bosutinib’s place in the evolving landscape of CML therapy, offering an alternative for CML patients across different treatment stages.

## Introduction

Chronic myeloid leukemia (CML) is a clonal hematopoietic stem cell (HSC) disease that accounts for approximately 30% of the incidence of adult leukemia (1). It is characterized by the presence of the Philadelphia (Ph1) chromosome, which results from a reciprocal chromosomal translocation between chromosome 22 and chromosome 9 [t(9;22)(q34;q11)] (2). This produces fusion of the *BCR* and *ABL1* genes into the pathogenic *BCR::ABL1* oncogene, generating a truncated hybrid protein with uncontrolled tyrosine kinase activity, which results in a proliferative advantage for mutant HSCs over normal HSCs (3). CML was classically classified into three stages: chronic (CP), accelerated (AP), and blast phase (BP) (4). Left untreated, the annual rate of disease progression to the advanced phases is about 10% (4). However, the risk of progression has been dramatically reduced with the introduction of tyrosine kinase inhibitor (TKI) therapy. As a result, the updated World Health Organization diagnostic criteria no longer recognize the AP, instead focusing on high risk features such as the presence of ABL1 kinase mutations and/or additional cytogenetic abnormalities (5). By contrast, the International Consensus Classification (ICC) maintains the traditional three-phase model for classifying CML (6).

Historically, the only curative therapeutic option for CML was allogeneic stem cell transplant (7). However, the CML treatment scenario was transformed by the development of TKIs. TKIs effectively block the interaction between the BCR::ABL1 oncoprotein and adenosine triphosphate (ATP), halting the cellular growth of the malignant clone. This “targeted” therapy altered the normal course of CML, raising the 10-year survival rate from 20% to 80%-90% (8, 9). The first-in-class TKI medicine licensed for the treatment of CML is imatinib mesylate more than 20 years ago (10). Nevertheless, some patients experienced treatment resistance or intolerance with imatinib, resulting in therapy failure in approximately 30%–35% of cases (11). Consequently, second- and third-generation TKIs such as dasatinib, nilotinib, bosutinib, and ponatinib were developed (12).

Current guidelines recommend a TKI (imatinib, dasatinib, nilotinib or bosutinib) as the frontline therapy for CML, while imatinib being the most commonly utilized, particularly in patients with comorbidities (11, 13, 14). Second-generation TKIs have been proposed for patients requiring a second line of treatment. While available evidence shows similar efficacy in comparison to imatinib, it is important to note that no head-to-head trials comparing second generation TKIs have been conducted to date (15). Therefore, the choice of therapy in this scenario is determined by the patient’s specific condition, particularly the existence of comorbidities, management of the side effects associated with each TKI, and how they impact the patient’s quality of life.

Bosutinib (also known as SKI-606) is an oral, second-generation TKI that binds to the kinase domain of BCR::ABL1 (16). Preclinical studies indicate that over the oral dosage range of 200-800 mg, it induces dose-proportional increases in plasma concentration and Cmax (17, 18). In addition, it has 34% absolute bioavailability in healthy people with a single 500-mg dose (17). Bosutinib inhibits 16 of 18 imatinib-resistant forms of BCR::ABL1 kinase in murine models (16). In preclinical safety trials, bosutinib had no effect on respiratory function or blood pressure, and had no/minimal effect on cardiac function (16, 19). It has also been shown to be non-mutagenic, non-carcinogenic, non-phototoxic, and non-clastogenic (16, 19). Some reproductive and developmental damage, as well as impaired fertility, was observed following bosutinib treatment in studies in rats and rabbits (16, 19).

Bosutinib 400 mg once daily (QD) is authorized for the treatment of individuals with newly diagnosed CP Ph1 CML, and 500 mg QD is approved for patients with CP, AP, or BP Ph1 CML who have developed resistance or intolerance to prior treatments (16). Since bosutinib was approved for the treatment of CML in 2012, numerous additional studies have been conducted, generating new data on its real-world and population-specific performance. In this review, we compile the latest available data on the efficacy and safety of bosutinib in the treatment of CML, in order to identify the patient profile that might potentially benefit more from this medication.

## First-line CML treatment with bosutinib

Approval of bosutinib as a first-line therapy for CML was based on the results of two pivotal phase 3 studies (summarized in **Table 1**). The BELA trial results were first published in 2012 and compared bosutinib 500 mg QD to imatinib 400 mg QD (15, 20). Although this trial did not meet its primary objective (complete cytogenetic response, CCyR, at 12 months), with a CCyR rate of 70% for patients on bosutinib vs. 68% for imatinib, it found a significantly higher major molecular response (MMR) rate (41% vs. 27%), faster times to CCyR and MMR, and a trend toward fewer on-treatment transformations to AP/BP with bosutinib compared to imatinib (20). The BELA trial also reported Kaplan-Meier estimates of overall survival at 12 months, which were greater than 99% in the bosutinib arm and 97% in the imatinib arm. Some factors might potentially have influenced the primary outcome of this trial, most notably the higher dose administered, which resulted in high rates of adverse event (AE)-related dose interruptions during the first few months (61% vs. 42%) and discontinuations (19% vs. 6%) in the bosutinib group versus imatinib group. The safety profile of bosutinib in this trial was consistent with previous reports and no new safety signals were identified (15).

**Table 1:** Summary of major findings from phase III studies of bosutinib as first-line therapy for patients with newly diagnosed CP-CML.

BELA trial				
		Bosutinib 400 mg QD	Imatinib 500 mg QD	*p-value*
12 months	Efficacy findings (21)			
	MMR rate	41.0%	27.0%	< 0.001
	CCyR rate	70.0%	68.0%	0.601
	Confirmed CHR	71%	85%	>0.999
	OS	>99%	97%	
	Dose escalation due to suboptimal response	4%	12%	
	Safety findings (21)			
	Frequent AEs Diarrhea Anemia Thrombocytopenia Hypophosphatemia	68% 81% 66% 47%	21% 84% 63% 63%	
	Discontinued treatment	19%	6%	
	Dose interruption due to AEs	61%	42%	
	Dose reduction due to AEs	39%	18%	
24 months	Efficacy findings (40)			
	Cumulative CCyR rate	79%	80%	
	MMR	47%	41%	
	Dose escalation due to suboptimal response	6%	18%	
	Survival rate	97%	95%	
	Safety findings (40)			
	Frequent AEs Diarrhea Nausea Anemia Thrombocytopenia Hypophosphatemia	70% 32% 25% 28% 8%	25% 36% 22% 28% 18%	
	Discontinued treatment	25%	9%	
	Dose interruption/reduction due to AEs	66%	45%	
	Dose reduction due to AEs	43%	21%	

BFORE trial				
		Bosutinib 400 mg QD	Imatinib 400 mg QD	*p-value*
12 months	Efficacy findings (22)			
	MMR rate	47.2%	36.9%	0.02
	CCyR rate	77.2%	66.4%	0.0075
	Survival rate	99.6%	97.9%	–
	Dose escalation due to suboptimal response	17.2%	27.5%	–
	Safety findings (22)			
	Frequent AEs Diarrhea Nausea Thrombocytopenia Muscle cramps	70.1% 35.1% 35.1% 2.2%	33.6% 38.5% 19.6% 26.4%	
	Discontinued treatment	22.0%	26.8%	
	Dose reduction due to AEs	56.3%	35.8%	
24 months	Efficacy findings (23)			
	MMR rate	67.2%	57.5%	0.02
	Cumulative CCyR	82.5%	76.8%	0.113
	OS	99.2%	97.0%	
	Dose escalation due to suboptimal response	20.1%	30.9%	
60 months	Efficacy findings (24)			
	Cumulative MMR rate	73.9%	64.6%	
	Cumulative MR rate	58.2%	48.1%	
	Safety findings (24)			
	Any grade treatment-emergent AEs (grade 3/4)	98.9%	98.9%	
	Frequent AEs Diarrhea Nausea Thrombocytopenia Muscle cramps	75.0% 37.3% 35.8% 3.7%	40.9% 42.3% 20.0% 30.6%	

AE, adverse event; CCyR, complete cytogenetic response; CHR, complete hematologic response; CML, chronic myeloid leukemia; MMR, major molecular response; OS, overall survival.

The results of the BFORE trial comparing bosutinib 400 mg QD to imatinib 400 mg QD were first published in 2018 (21). At 12 months, patients who received bosutinib had substantially greater rates of MMR and CCyR than patients who did not receive bosutinib (MMR: 47% vs. 37%; CCyR: 77% vs. 66%, respectively). Furthermore, as compared to imatinib-treated patients, bosutinib demonstrated quicker responses at 24 months (**Table 1**) (22). The safety findings were consistent with prior studies, and no new safety signals were identified. Long-term efficacy and safety of bosutinib were demonstrated in the final analysis of the BFORE trial after 5 years of follow-up (23). Bosutinib was superior to imatinib in terms of MMR rates (47.4% vs. 36.6%; 1.57 [1.11–2.22]) (23). This data was also confirmed in the Japanese population (24). Regarding its safety profile, both bosutinib and imatinib treatment showed an increase in the incidence of cardiac, effusion, renal, and vascular treatment-emergent adverse events (TEAEs) after 5 years of follow-up, but no new safety signals were observed (23). Overall survival rates in the BFORE study demonstrated the long-term efficacy of bosutinib. After 5 years of follow-up, both bosutinib and imatinib had excellent overall survival rates (94.5% and 94.6%, respectively), highlighting the effectiveness of bosutinib in producing long-term responses.

These data support the use of bosutinib 400 mg QD in newly diagnosed CP-CML patients. In current practice, first-line treatment is determined on the basis of the estimated risk-benefit ratio in each particular patient. Although no direct comparisons of bosutinib vs. dasatinib or nilotinib have been made, indirect comparisons have shown that bosutinib is as effective as nilotinib and dasatinib in the treatment of individuals with newly diagnosed CP-CML (25). Thus, in those patients considered candidates for treatment with second-generation TKIs, the choice is determined by the patient’s comorbidities and drug safety profile. The selection of second-generation TKIs may also be influenced by the availability of dasatinib as a generic medication. Treatment with dasatinib or nilotinib is not indicated in individuals with significant cardiovascular risk, so bosutinib is a better option since it has a better cardiovascular profile (26). On the other hand, patients who have had or are having gastrointestinal (GI) issues or hepatotoxicity for any reason would not be good candidates for bosutinib treatment.

## Later-line CML treatment with bosutinib

### Second-line treatment

Bosutinib is also approved for patients with Ph1 CML who are resistant/intolerant to prior therapy (16). The approval of bosutinib 500 mg QD for patients with CML who were previously treated with ≥1 TKI was based on the results of a multicenter, open-label phase 1/2 study that investigated bosutinib’s performance in patients with imatinib-resistant or -intolerant CML. The study had distinct cohorts for CP-, AP-, and BP-CML (27–29). Most of the participants in the study (69%) were patients with imatinib-resistant CP-CML (27–29). Cumulative major cytogenetic response (MCyR) and CCyR rates (newly acquired or maintained from baseline) were 60% and 50%, respectively, by year 5 (29). The Kaplan-Meier probability of maintaining an MCyR or CCyR response after 5 years was 71% and 69%, respectively (29). Most AEs occurred within the first 2 years (29). Overall, GI disturbances were the most prevalent (diarrhea 86%, nausea 46%, and vomiting 37%), with thrombocytopenia (25%) being the most common grade 3/4 toxicity (29). From years 3 to 5, none of the four on-treatment reported fatalities were linked to bosutinib. In summary, bosutinib demonstrated long-term efficacy and a tolerable safety profile in patients with CML treated for up to 5 years in the second-line setting (27–29). A summarized comparison of the efficacy of other second-generation TKIs (dasatinib and nilotinib) after imatinib failure at 24 months is shown in **Table 2**. In general, the three second-generation TKIs exhibited comparable efficacy, with probabilities of achieving CCyR around 50% (28, 30, 31).

**Table 2:** Efficacy of second-generation TKIs after imatinib failure.

TKI	Dasatinib (32)		Nilotinib (31)		Bosutinib (29)	
Dose/Frequency	100 mg/BID		400 mg/BID		500 mg/QD	
	Resistance	Intolerance	Resistance	Intolerance	Resistance	Intolerance
Follow-up time	24 months		24 months		24 months	
CCyR^a^	44%	67%	41%	51%	48%	52%
MMR^b^	37%	–	28%	–		
PFS^c^	80%		64%		79%	
OS^d^	91%		87%		92%	

MCyR, major cytogenetic response; MMR, major molecular response; OS, overall survival; PFS, progression-free survival; QD, once daily. ^a^: Complete cytogenetic response. ^b^: major molecular response. ^c^: progression-free survival. ^d^: overall survival.

The efficacy and safety of bosutinib 500 mg QD was further evaluated in patients with CML who had previously failed TKI therapy and were otherwise ineligible for other TKIs in the post-approval phase 4 BYOND trial (32, 33). This was a single-arm, open-label, non-randomized study assessing the efficacy of second-, third-, and fourth-line bosutinib treatment (32, 33). To be considered a responder, the patient must have had maintenance of baseline response for ≥52 weeks for cytogenic response or an improvement from baseline (32, 33). Bosutinib was able to induce a cumulative MMR rate by 1 year of 70.5% in the overall Ph1 CP-CML cohort (32). After 3 years of follow-up, bosutinib continued to show efficacy in previously treated patients with Ph1 CP CML, with 71.8% of patients achieving or maintaining MMR (33). Among responders, the estimated probability of maintaining CCyR, MMR, or deep molecular response (DMR) was 96.5%, 87.2%, and 80.7%, respectively (33).

While there are no head-to-head comparative trials between bosutinib and other second-generation TKIs in the second- and later-line settings, a matching-adjusted indirect comparison analysis in patients receiving second-line treatment for CP-CML found that bosutinib had a longer progression-free survival (PFS) (hazard ratio 0.63) and overall survival (OS) (hazard ratio 0.82) than either dasatinib or nilotinib (34). However, it is worth noting that dasatinib and nilotinib showed higher rates of MCyR than bosutinib (34).

### Third- and later-line treatment

The safety and efficacy of bosutinib 500 mg QD in the setting of third-line CP-CML treatment was evaluated in patients who had previously been treated with imatinib, followed by dasatinib and/or nilotinib (35, 36). This phase 1/2 study included 118 individuals, most of them in the third-line setting (n=115) and the rest in the fourth-line setting (n=3). Patients were followed up for a median of 28.5 months and responses were divided according to resistance/intolerance to previous treatments (35). Among these patients, 32% achieved MCyR and 24% attained CCyR. Notably, bosutinib treatment resulted in CCyR in 1 out of 3 patients who had failed to respond to three prior TKIs (35). Patients with dasatinib intolerance (76%) and nilotinib resistance (86%) had a higher probability of maintaining MCyR at 2 years, compared to those with dasatinib resistance (34%) and nilotinib intolerance/prior treatment with all three TKIs (50%) (35). The 2-year estimated PFS was 73%, and estimated OS was 83% (36). The safety profile of bosutinib was deemed acceptable; the majority of TEAEs were GI issues of mild to moderate severity and rash (35). Long-term efficacy and tolerability results of this trial were consistent, demonstrating durable efficacy and a toxicity profile similar to previous bosutinib studies (36).

Regarding the specific findings of the BYOND trial, cumulative CCyR rates by 1 year were 83.9% and 73.3% for patients in the third-line and fourth-line settings, respectively (32). Cumulative MMR rate by 1 year was 74.5% for the patients in the third-line setting, and 56.3% for those in the fourth-line setting (32).

ASCEMBL is a phase 3, open-label, randomized study of asciminib vs. bosutinib in CML after two or more prior TKIs (37). In this study, asciminib demonstrated superior efficacy vs. bosutinib. One striking aspect was that bosutinib outcomes in ASCEMBL were worse than in the prior BYOND study (28, 29). Two factors could explain these findings. First, there was a higher proportion of resistant patients in the bosutinib arm; and second, patients failing bosutinib treatment due to a lack of efficacy were offered the possibility of switching to asciminib. However, patients who discontinued treatment with bosutinib because of intolerance (or any reason other than lack of efficacy) were not allowed to switch to asciminib. This might have impacted negatively on the percentage of patients who discontinued bosutinib.

Understanding their different modes of action is important for comprehending the efficacy and toxicity profile of each medication. Bosutinib is a second-generation TKI that inhibits the BCR::ABL1 protein by binding to its ATP-binding site, thereby blocking its tyrosine kinase activity (38). In contrast, asciminib is a first-in-class STAMP (Specifically Targeting the ABL Myristoyl Pocket) inhibitor that binds to the myristoyl pocket of the BCR::ABL1 protein, leading to its inactivation through an allosteric mechanism (39). The binding of asciminib outside of the ATP site may account for the greater efficacy demonstrated by this agent in the ASCEMBL study.

In our opinion, although asciminib has been shown to provide superior results, bosutinib remains a valid option in patients failing second-generation TKIs, with a high likelihood of treatment success, especially in cases with intolerance to prior TKIs. In patients who have previously been intolerant to TKIs, bosutinib may be a better alternative than asciminib due to its established safety profile, which showed a lower incidence of cardiovascular events in the ASCEMBL trial. This makes it a potential option for people who have pre-existing cardiovascular issues or are at high risk of such complications. Furthermore, while asciminib has shown higher effectiveness, bosutinib may still be useful in individuals who have developed resistance to several second-generation TKIs. The choice of treatment may be determined by the specific mutation profile and prior response patterns. Furthermore, due to its demonstrated efficacy in eliciting rapid cytogenetic and molecular responses, bosutinib may be considered in situations requiring a prompt treatment response. Of note, in the ASCEMBL study, bosutinib showed fewer cardiovascular events than asciminib, a topic that will be further explored in the management of AEs section of this review.

### Advanced phase CML

A subanalysis of the phase 1/2 open-label study evaluating the efficacy and safety of bosutinib as second-line therapy in CP-CML patients evaluated for the first time the durability of response and long-term safety of bosutinib in the fully enrolled advanced leukemia cohort (AP-CML, BP-CML, and Ph1 acute lymphoblastic leukemia {ALL]) during a follow-up of up to 4 years (40). Bosutinib demonstrated a durable response among the 167 patients included in the study, with 50% of AP responders sustaining an MCyR at 4 years (40). Furthermore, 25% of BP responders sustained their response to therapy after 1 year. Despite the small number of ALL patients that were included, some of them responded (40, 41).

Another study looked at the safety of combining inotuzumab ozogamicin (an antiCD22 recombinant humanized monoclonal antibody) with bosutinib as treatment for lymphoid BP-CML (42). The trial studied the role of this combination with three dose levels of bosutinib (300 mg/d, 400 mg/and 500 mg/d) in 18 patients with a median follow-up of 44 months. The maximum tolerated dose of bosutinib was 400 mg daily and dose limiting toxicities included grade 3 skin rashes (42). CMR was seen in 56% of patients (42). Thus, this drug combination resulted in high rates of morphologic and molecular response with an acceptable toxicity profile in this population.

## Real-world effectiveness and safety data on bosutinib treatment

Several reports on the real-world use of bosutinib have been published since its marketing approval (**Table 3**). The efficacy and safety of real-life bosutinib administration were retrospectively investigated in a cohort of 85 Italian patients who were resistant, refractory, or intolerant to multiple TKIs (43). The median age of this group was 60 years, as reported by Attolico et al. in 2018 (43). MMR/DMR was achieved by 46% of evaluable patients (43). In terms of safety, 88% of patients completed the study after a median follow-up of 26 months (range: 3-49 months). Fourteen patients (16%) experienced hematological toxicity, while 48% experienced extra-hematological toxicity. Dose reductions were required in four patients (5%) due to side effects, with diarrhea being the most common (43). Another study conducted in the United Kingdom and the Netherlands found that bosutinib, when used in routine clinical practice in CML patients in the third and fourth lines of treatment, achieved high rates of cumulative CCyR and MMR similar to those observed in clinical trials (67% and 55%, respectively) (44, 45). Remarkably, despite the fact that most patients had preexisting comorbidities, bosutinib was generally well tolerated, with the majority of patients not experiencing grade 3/4 AEs (45). The real-world performance of bosutinib in fourth-line treatment was also assessed in a Spanish cohort (46). Sixty-two patients with CP-CML who had been previously treated with imatinib, nilotinib, and dasatinib were retrospectively reviewed (46). The probability of either maintaining or improving CCyR was 25% in the subgroup of patients without CCyR at baseline and 94% in patients with CCyR at baseline (46). The probability of achieving MMR in patients who did not respond at baseline was 14% or 42% in patients with or without CCyR at baseline, respectively. Additionally, bosutinib had an acceptable safety profile in patients who had been intolerant to prior TKI therapy (46).

**Table 3:** Summary of effectiveness and safety results in a real-world setting after bosutinib treatment in CML patients.

Study	Design	N	Key effectiveness outcomes	Key safety outcomes
Claudiani et al., 2022 (43)	Multicenter retrospective, non-interventional study in CP-CML patients	87	- Cumulative CCyR 67% - Cumulative MMR 55%	- Discontinuation due to - Lack of efficacy/disease progression:17% - Adverse events: 38% - ≥1 adverse event: 94%
Latagliata et al., 2021 (42)	Multicenter retrospective, non-interventional study in CP-CML patients >65 years old	101	- CCyR: 77.0% - MR: 66.6% - MMR: 21.8%	- AEs - Grade 3/4 hematological toxicity: 6.9% - Grade 3/4 extra-hematological toxicity: 18.8%
García-Gutiérrez et al., 2019 (45)	Multicenter retrospective, non-interventional study in Spanish CML patients	62	- CCyR: 25% - MMR: 24%	Discontinuation due to - Intolerance: 16% - Lack of efficacy 10% - AEs - Diarrhea: 39%

AE, adverse event; CCyR, complete cytogenetic response; MR, molecular response; MMR, major molecular response.

In summary, findings from clinical trials and real-world evidence indicate that bosutinib is a viable therapeutic option for CP-CML patients who are resistant/intolerant to multiple TKIs.

## Bosutinib management

Multiple studies on bosutinib dose optimization in CML patients have been reported (**Table 4**). In frontline treatment of CP-CML, an analysis including patients participating in the BFORE trial who had a bosutinib dose reduction to 300 mg for toxicity-related issues (or to 200 mg QD for four weeks maximum, after which dose escalation or treatment discontinuation was required) (47) evaluated the effectiveness of a lower dose of bosutinib. Thirty-one percent of the patients in the bosutinib arm had their dosage reduced to 300 mg QD (without further reduction) and 12% to 200 mg (47). Among patients who had a dose reduction to 300 mg QD, 62% had maintained MMR for >6 months afterwards, in comparison to 78% of the patients who remained on 400 mg achieving MMR (47). In fact, 17% of patients who reduced the dose to 300 mg QD maintained MMR before and after dose reduction, and 45% of them achieved MMR for the first time after decreasing the dose (47). In patients who had their bosutinib dosage reduced to 200 mg QD, 36% obtained MMR >6 months later, 21% maintained MMR before and after the decrease, and 15% achieved MMR for the first time after the dose was lowered (47). At the safety level, the incidence of diarrhea, thrombocytopenia, nausea, vomiting, and anemia decreased by more than 10% in individuals whose bosutinib dosage was reduced to 300 mg QD or 200 mg QD, with additional decreases in abdominal pain, fatigue, and rash after dose reduction to 200 mg QD (47). Thus, the study showed that management of AEs through bosutinib dose reduction enabled patients to continue on treatment, maintaining its efficacy while improving tolerability.

**Table 4:** Summary of dose optimization studies with bosutinib.

Line	Study	Setting	Number of patients	Outcomes
First line	BFORE subanalysis (46)	Newly diagnosed CP-CML patients received an initial dose of bosutinib of 400 mg QD. Some of them had a dose reduction because of toxicity-related issues to 300 mg (or to 200 mg QD for 4 weeks maximum after which dose escalation or treatment discontinuation was required). Response at all assessments >6 months after the first bosutinib dose reduction to 300 mg QD or 200 mg QD.	268	Efficacy - CCyR >6 months after dose reduction to 300 mg QD: 68.4% - CCyR >6 months after dose reduction to 200 mg QD: 46.9% Safety - Incidence of diarrhea, thrombocytopenia, nausea, vomiting, and anemia decreased >10% among patients with bosutinib dose reductions to 300 mg QD or to 200 mg QD.
Second and later lines	BODO study (48)	Bosutinib was commenced with 300 mg QD and was (in the absence of >G1 toxicities) dose-increased by increments of 100 mg daily dosing every 14 days if applicable up to a maximum dose of 500 mg QD.	57	Efficacy (24 months) - MMR: 79% - MMR in patients refractory to previous therapy and not in MMR at baseline: 64% Safety - Rate of GI-toxicity (grade 2 to 4) within the first 6 months of treatment was 60.0% (95% CI: 45.2% to 73.6%).
Second line	BEST study (49)	Patients with CML ≥60 years were given 200 mg of bosutinib for 2 weeks before increasing the dosage to 300 mg. In the absence of meaningful toxicity, patients with BCR::ABL1 transcript at 1% or lower stayed on 300 mg, while patients with transcript > 1% increased to 400 mg	63	Efficacy (36 months) - MMR: 78% - DMR: 46% - OS: 81% Safety (36 months) - Discontinued treatment: 43%

CCyR, complete cytogenetic response; DMR, deep molecular response; GI, gastrointestinal; MR, molecular response; MMR, major molecular response.

More recently, pharmacokinetic population modeling was conducted to quantify the impact of reducing the initial dosage of bosutinib from 500 to 400 mg QD (48). The analysis integrated data from the BELA and BFORE studies and from trial B1871048 (a phase 2 study of first-line bosutinib in CP-CML in a newly diagnosed Japanese cohort).The exposure-response efficacy analysis revealed that cumulative MMR and CCyR were comparable in patients in the BFORE and B1871048 trials who had a starting dosage of 400 mg QD and those in the BELA trial who were given a starting dose of 500 mg QD (48). Additionally, in terms of safety, patients in the BFORE and B1871048 trials who received a starting dose of 400 mg QD had a lower rate of permanent discontinuation due to AEs (48). Overall, the results suggest that a bosutinib starting dose of 400 mg QD improves tolerability without affecting the efficacy in newly diagnosed adult CP-CML patients.

Another trial investigating the effect of a bosutinib step-in dosing regimen in reducing GI toxicity was focused in patients with CML after failure or intolerance to other second-generation TKIs (49). In this phase 2 trial, the “Bosutinib Dose Optimization Study” (BODO; NCT02577926), the bosutinib starting dose was 300 mg QD and was increased by increments of 100 mg daily dosing every 14 days in the absence of grade 1 or higher toxicities up to a maximum dose of 500 mg (49). Although the advantage of the step-in dosing concept in lowering the frequency of GI toxicity could not be demonstrated because of incomplete recruitment, this regimen of bosutinib administration, with a mean bosutinib dosage of 403 mg/day, induced optimal responses in 64% of patients refractory to previous therapy and not in MMR at baseline, while GI toxicity rarely led to treatment discontinuation (49).

A subanalysis of the BEST study sought to determine the effectiveness of initial low-dose bosutinib in elderly CML patients in the second-line setting, considering that the fixed starting dosage of 500 mg QD may still be too high for this subpopulation (50). The study was designed so that participants over the age of 60 were given 200 mg of bosutinib for 2 weeks before increasing the dosage to 300 mg (50). In the absence of meaningful toxicity, patients with *BCR::ABL1* transcript at 1% or lower stayed on 300 mg, while patients with transcript >1% increased to 400 mg (50). The study revealed that the likelihood of reaching or maintaining MR by 36 months was 78% (36). Moreover, the majority of patients stayed on 300 mg or less of bosutinib (50).

### Toxicity management

The appropriate management of low-grade persistent AEs is becoming increasingly important in CML patients, particularly given the possibility of lifetime TKI therapy. However, data is limited as regards the timing and management of AEs associated with long-term TKI therapy.

The low incidence of adverse effects related to bosutinib treatment is worth noting, although GI AEs, specifically diarrhea, are expected. The incidence of moderate-grade adverse effects has not exceeded 20% in clinical trials and in routine clinical practice, particularly within the first 12 months of treatment initiation (51).

Although diarrhea is frequent in CML patients taking bosutinib as second- or later-line therapy (around 85% of patients) (51), the prevalence of grade 3/4 diarrhea episodes is lower (approximately 9.5% of patients) (36, 52). Nausea, vomiting, and abdominal discomfort are also among other GI TEAEs associated with bosutinib (36, 52). It is recommended that all patients receiving bosutinib be assessed for diarrhea and signs of dehydration (53, 54). Non-pharmacological treatment options include dose reduction, increased fiber consumption, and abstaining from foods or ingredients that can cause loose stools (such as alcohol, lactose-containing products, laxatives/stool softeners, raw fruits and vegetables, spicy or fatty meals, caffeine, etc.) (51). Proton pump inhibitors should be avoided due to the potential reduction in bosutinib exposure (19). Pharmacological therapies include mostly drugs that reduce stool discharge, such as antidiarrheals; antiemetics and/or fluid replacement are also used (51). In practice, the majority of affected individuals are treated for diarrhea with concomitant antidiarrheal drugs, most often loperamide (51).

Studies concerning the concomitant administration of symbiotics to mitigate the incidence of GI disorders remain limited in the scientific literature. However, a preliminary exploration conducted in a small-scale pilot study (55) detailed the clinical observations derived from the treatment of 35 patients with bosutinib concomitantly supplemented with a symbiotic regimen. Notably, the findings demonstrated a statistically significant reduction in GI adverse effects associated with bosutinib administration (55). Furthermore, the symbiotic intervention exhibited a beneficial impact on treatment adherence and quality of life parameters pertaining to GI health (55). While further research is needed, these findings suggest the potential of symbiotics in mitigating bosutinib-induced GI complications, offering avenues for improved patient outcomes and therapeutic compliance.

Hepatotoxicity TEAEs are seen in around 25% of patients receiving bosutinib as a second-line therapy, with occurrence of grade 3/4 hepatotoxicity, specifically increased alanine and aspartate aminotransferase (ALT/AST) levels observed in 11% of patients (51). Transient dosage pauses in 37% of patients, dose reductions, or concomitant medicines were used to manage patients with high ALT/AST levels (51). These TEAEs had an average duration of 26 days, indicating that bosutinib hepatotoxicity can be reversed with a change in dosage or a temporary discontinuation in therapy. Of note, 74% of patients who resumed bosutinib after ALT/AST elevations did not experience additional ALT/AST events (51). However, permanent discontinuation should be considered if the patient does not respond to dose adjustment or continues.

Cardiac and vascular AEs are infrequent with bosutinib, as only 3.9% of patients have been reported to develop grade III-IV cardiovascular toxicities (56). In the ASCMBL trial comparing asciminib with bosutinib, a higher number of patients developed cardiovascular disease in the asciminib arm (3.2%) compared with the bosutinib cohort (1.3%) (37). Furthermore, these incidence rates seem to be lower than other second-generation TKI historic data (56). Dose interruption has been demonstrated to be effective in the management of those patients who develop cardiac TEAEs (51). Indeed, the majority of the patients whose bosutinib was interrupted were successfully retreated (56). Concomitant medication (47%) or dose reduction (5%) were other management strategies (51).

Regarding hematological toxicities, thrombocytopenia is the most frequently reported (around 40% of patients) and the most common reason for temporary treatment discontinuation or dosage reduction (15, 51).

Bosutinib has been associated with pulmonary side effects, such as pleural effusion (PE), occurring at a rate of 4% to 8% in long-term safety trials (27, 28, 41). Interestingly, it has been shown that, in certain individuals, bosutinib might worsen dasatinib-induced pulmonary arterial hypertension and PE (57). However, a recent retrospective study found that 70% of patients who were treated with bosutinib in second or subsequent lines after having one or more PE events while on dasatinib therapy did not develop PE during the study treatment (58), suggesting that bosutinib may be a valid therapeutic choice in CML in a selected population of patients who developed PE while on dasatinib treatment.

In the context of renal dysfunction, the prevalence of elevated blood creatinine levels, irrespective of severity, among patients in the CP second-line cohort undergoing bosutinib treatment was increased during years 3 and 4 compared to the initial 2 years (36, 52). Similarly, within the CP third-line cohort, the incidence was notably elevated in the fourth year relative to preceding years (36, 52). Nevertheless, long-term administration of bosutinib appears to induce a reversible decline in renal function, demonstrating a frequency and characteristics akin to those observed with prolonged imatinib use (59). Nevertheless, optimal management necessitates vigilant patient assessment for manifestations of renal dysfunction, such as alterations in urinary frequency, polyuria, or oliguria (51). Continuous monitoring of renal function, especially in patients with preexisting renal impairment or predisposing factors for renal dysfunction should be performed (51). The recommended starting dose of bosutinib varies based on creatinine clearance (CrCL) levels: for CrCL 30–50 ml/min, the recommended starting dose is 400 mg/day, while for CrCL <30 ml/min, the starting dose is reduced to 300 mg/day (51).

## Bosutinib treatment in special situations

### Pregnancy

TKI therapy should be discontinued in case of pregnancy (19). Despite this general recommendation, some patients require treatment with a TKI during pregnancy. Clinical data has been reported mostly with imatinib, mainly in the third trimester. As regards experience with bosutinib in this setting, despite preclinical studies showing reproductive toxicity (16, 19), clinical data on the risk of teratogenesis or miscarriage is scarce. In clinical practice, a recent report including 33 pregnancies noted that 15 of them (45.5%) resulted in healthy newborns, and nine resulted in abortions, although none of these were considered by the healthcare provider to be related to bosutinib treatment (60). Moreover, there is also a risk of diminished fertility (female/male) with bosutinib, according to preclinical studies (16, 19). Female patients should have a pregnancy test before beginning bosutinib medication, and adequate contraception should be used during treatment and for ≥1 month after the last dose to avoid becoming pregnant while receiving bosutinib (16). Patients should also be informed about the risks of using bosutinib during pregnancy (19). For male patients, there are no current restrictions. Despite limited published experience, no association has been identified between men on bosutinib treatment and the occurrence of miscarriages in their partners or malformations in full-term pregnancies (60). On the other hand, although there are safety reports on the use of imatinib in the third trimester, the National Comprehensive Cancer Network and European Leukemia Net guidelines clearly advise against using any TKI during pregnancy (53, 54).

### Pediatric patients

Imatinib is the first-line treatment recommended in pediatric CML patients (61). However, 30% of children have suboptimal response to imatinib (61). Bosutinib is not currently licensed for use in children in Europe, although it has been recently approved in the USA for use in children starting from the age of 1 year (62). Pediatric patients who are intolerant or do not respond to imatinib or second-generation TKIs may benefit from the approval of bosutinib. A phase 1/2 study of bosutinib in 23 patients with newly diagnosed or resistant/intolerant Ph1 CML (63) established 300 mg/m^2^ QD as the recommended phase 2 dose (63). For resistant or intolerant individuals, 400 mg/m^2^ QD provided exposure equivalent to 500 mg QD in adults, with comparable preliminary effectiveness to other second-generation TKIs (63).

### Drug-drug interaction of bosutinib

Drug interactions also need to be considered when choosing a TKI in CML patients. Bosutinib has been shown to have a particular interaction profile (64). All second-generation TKIs block the BCR::ABL1 tyrosine kinase, and are often non-selective medications that can inhibit other tyrosine kinases, such as cKIT or PDGFR (64). These medications are taken orally and have intermediate bioavailability, a large volume of distribution, strong protein binding, and elimination following extensive metabolism involving different cytochrome P450 (64). Because all BCR::ABL1 inhibitors are cytochrome P450 substrates, their rate of elimination can be influenced by other medications that enhance or decrease the activity of these isoenzymes. Furthermore, dasatinib, imatinib, nilotinib, and ponatinib control CYP450 interaction by inhibiting the enzyme activity of several of the isoenzymes (64). Bosutinib does not share this property, suggesting that it might be a good treatment option for patients who are polymedicated as it prevents possible drug-drug interactions, although no specific recommendations have been published in this regard.

### Treatment discontinuation

A retrospective review of CP-CML patients receiving bosutinib or ponatinib who discontinued treatment in a real-life single-center study in the USA showed that among the eight patients who discontinued bosutinib (65), safety was similar to discontinuation with other TKIs. Patients who had lost MMR responded after restarting treatment (65). In Spain, a discontinuation study (BOSUTRF) is ongoing.

### Elderly population

As previously mentioned, both controlled trials [BEST study (50)] and real-world data (43) show that bosutinib is effective and safe in older patients with significant comorbidities and resistance or intolerance to earlier TKIs. Efficacy results are comparable to those in younger patients, with a reported CCyR of 77% in the real-world setting (43). Tolerability in the elderly is also comparable to the younger population (43). Nevertheless, the use of bosutinib in this age group depends on various factors: comorbidities, other drug interactions and supportive care. Ultimately, the use of bosutinib in older adults is determined on a case-by-case basis and should be evaluated by the patient’s healthcare team.

## Discussion: Recommendations for bosutinib treatment in CML patients

Based on data reviewed in the present document, our conclusions on the use of bosutinib in clinical practice are as follows:

- Among second-generation TKIs, bosutinib could provide a clinical advantage in patients with cardiovascular risk factors.
- Long-term data demonstrate the safety of bosutinib in this setting.
- In cases of resistance or intolerance to imatinib, bosutinib has shown similar efficacy to other second-generation TKIs, with an optimal safety profile and low incidence of adverse events.
- In third-line treatment, bosutinib has proven effective in sustaining previous responses in patients who discontinued treatment due to intolerance following prior second-generation TKI failure.
- Bosutinib has proven to be effective in older individuals. Due to its favorable drug interaction profile, bosutinib is an adequate therapeutic option for patients with multiple medications.
- As bosutinib does not contain lactose in its formulation, it should be recommended in patients with lactose intolerance.
- The recommended dosage for first-line treatment is 400 mg, while for second and subsequent lines, it is 500 mg. If intolerance is the reason for therapeutic failure, doses lower than 500 mg are recommended from treatment initiation. In certain cases, especially in older patients, starting with 300 mg and escalating to 400 mg may be considered to improve drug tolerance and achieve favorable molecular responses.
